# Supplementary material for: Are carbon emissions trading and green financial instruments synergistic? -Comprehensive quantitative research based on content analysis
Source: PLoS One. 2024 Mar 7;19(3):e0298601. doi: 10.1371/journal.pone.0298601 (PMC10919601; doi:10.1371/journal.pone.0298601)
Supplement: S1 Data — (ZIP) [file pone.0298601.s001.zip › Data/地级市Z_F工作报告.zip!/║╙─╧/░▓╤⌠╩╨/└┤╫╘╣1⁄2╓┌║┼í╛┬φ┐╦╩2╛▌═°í┐_2015.docx]

各位代表

现在，我代表市人民政府，向大会报告工作，请予审议，并请各位政协委员提出意见。

一、2014年工作回顾

2014年，面对极为错综复杂的形势和艰巨繁重的任务，在省委、省政府和市委的正确领导下，全市上下认真贯彻落实党的十八大和十八届三中、四中全会精神，坚持转中求进、改革创新，努力保增长、调结构、促改革、惠民生、防风险，务实重干，锐意进取，经济社会持续稳定发展，在全面建成小康社会的道路上迈出了新步伐。

--综合实力持续增强。全市生产总值1593.2亿元，增长8.7%，人均生产总值达到4万元。一般公共预算收入95.3亿元、支出192.1亿元，分别增长10.9%和7.5%。

--转型升级持续加快。谋划实施工业转型升级项目95个、战略性新兴产业项目95个，高新技术产业增加值176亿元，增长19%;服务业增加值524.3亿元，增长7.6%。产业结构不断优化，三次产业比达到9∶58∶33，第三产业占比提高1.1个百分点。11家企业入围河南省百强、百高企业。

--关键改革持续深化。启动两批78项经济体制改革任务，完成45项。清理行政审批和非行政许可事项150个。整合行政审批和服务事项416个，入住职能部门、企事业单位64家，建成市民之家一站式综合服务平台。

--农业效益持续增长。全市高标准粮田面积达到90万亩，粮食总产220.3万吨，实现"十二连增"。农业产业集群总产值突破228亿元。现代农业示范园区面积达到76万亩。成功创建国家级蔬菜标准园9个、国家级标准化规模养殖场13个、省级农业标准化示范基地18个。

--开放招商持续提升。与中建材、国药集团、国旅集团、保利集团、华录集团等央企的战略合作进一步深化，开启央地合作新局面。国旅集团理事会第十次全会、九三学社中央安阳文化生态旅游高端会议在我市隆重召开。第六届航空运动文化旅游节成功举办。韩国来安游客突破12万人次。

--民生福祉持续改善。十项民生工程51件实事全面完成。在全省率先实施社会保障一卡通、城镇居民大病保险制度。新增公交车164辆。3条旅游专线、10辆双层旅游大巴开通运营。"安阳城市一卡通"成功发行。预计城镇居民人均可支配收入25089元，增长9%;农民人均纯收入10829元，增长12%。居民消费价格增长1.9%。

一年来，我们重点抓了以下六个方面工作:

(一)稳运行保增长，在复杂形势中推动经济平稳发展。面对经济下行和结构调整的双重压力，把保增长摆到更加突出的位置，分类施策，精准发力，有效促进了经济平稳运行、稳中有进。扩大有效需求。制定出台促进经济稳定增长的若干意见，加快实施政府投资计划，激发民间投资活力，争取中央和省补助资金17.3亿元加强重大项目建设，一批打基础、管长远的项目建成投用。201个省、市重点项目完成投资533.4亿元。全市固定资产投资1446.7亿元，增长18.5%;社会消费品零售总额525.3亿元，增长12.2%;进出口总额14.9亿美元。帮扶企业解困。针对冶金建材、煤化工等传统支柱行业经营困难问题，深入实施支持工业发展的39条意见，推动企业延链补链、降本增效，加快脱困转型。完善企业服务长效机制，积极组织产销对接、产用对接，最大限度地降低企业经营困难。规模以上工业企业利润总额211.6亿元，增长3.9%。强化要素保障。优化金融生态环境，建立债权银行联席会和贷款共进退机制，有效防范金融风险。搭建银企对接平台，发挥企业应急周转资金作用，帮助企业融资120.9亿元。全年金融机构新增贷款125.6亿元。规范涉企行政事业收费，落实小微企业税收政策。土地、人才、能源等要素得到较好保障。

(二)调结构促转型，在方式转变中提升发展质量效益。坚持扩大增量与调整存量并重，改造提升与培育重点并举，推动经济结构转型、发展方式转变。强力抓好工业转型。深入推进嫁接式转型开发，大力支持战略性新兴产业和高成长性制造业发展，加快推动传统产业改造升级，产业结构逐步改善，竞争力不断提升。安化保险粉、安钢冷轧酸洗轧机联合机组、光远新材料电子布等33个项目建成或投产。安钢冷轧连退镀锌生产线、江南装备制造产业园等54个项目开工建设。大通电子等一批战略性新兴产业和高成长性制造业项目快速推进。我市成功入选国家级工业电子商务区域试点。汤阴县荣获首批"中国食品工业名县"称号。加快服务业升级。把服务业作为弥补产业发展短板的重点，推动文化旅游、电子商务、商贸服务、健康服务、养老服务等高成长性服务业快速发展，新的消费热点和服务业增长点正在形成。全年接待国内外游客2968.9万人次，增长8.6%;旅游总收入197.5亿元，增长8.1%。万达广场主体竣工，义乌丹尼斯旗舰店、安阳物流港投入运营。国贸中心、悠乐生活广场等项目进展顺利。全市300亩以上的物流园区增至7个，300辆车以上的货运企业达到12家。超过2000平方米的商品市场达到85个。房地产开发投资161.3亿元，增长9.3%。三产对经济增长的贡献率达到28.7%。完善科学发展载体。加强产业集聚区基础设施建设，新建成标准化厂房141万平方米，要素吸附力、综合承载力不断增强。钢铁精深加工、装备制造、汽车零部件等5个主营业务收入超百亿元的特色产业集群纳入省产业集群培育工程。集聚区工业总产值1442亿元，占全市比重的44.4%。服务业"两区"规划全部获批，入住企业达到370家。红旗渠经济技术开发区成为全省唯一被列入国家循环化改造示范试点园区。加强科技创新。与合肥工业大学合作更加紧密。建成中科院创新育成分中心。新培育高新技术企业10家。省级以上研发中心增至112家。实施重大科技专项3个。国家级铁制品质检中心获批筹建。成功创建国家知识产权示范城市。强化环境保护。以壮士断腕、刮骨疗毒的决心和勇气，狠抓大气污染防治工作，大力淘汰落后产能，企业停产整治356家，取缔石灰、石料和"土小"企业173家，342台10吨以下燃煤锅炉全部拆除，大气环境质量得到改善。积极推进节能减排，成功入选国家首批新能源示范市。预计单位生产总值能耗下降4%。

(三)抓统筹促协调，在产城互动中加快城乡一体发展。着眼于城市空间拓展和产业布局优化，强化中心城区带动，新型城镇化的速度、水平和质量进一步提高。全市城镇化率突破50%。推动产城融合。加快完善城乡一体化示范区基础设施，增强综合承载能力。安阳精密制造产业基地、贯辰通用航空产业基地、国际汽博产业园等一批项目的实施，有效强化了产业支撑。羑河综合治理成效初显。提升城市功能。加强城市环境综合整治，打造"净、绿、亮、美、畅"宜居环境。太行路、朝霞路等7条"断头路"基本打通，3座人行天桥投入使用。争取中央和省补助资金7.6亿元，启动棚户区改造项目11个，17个棚改安置房项目全部开工。新增集中供热面积498万平方米、日污水处理能力25万吨。完善综合交通体系。晋豫鲁铁路安阳段通车运营。安阳机场预可研报告通过省政府审批。安鹤快速通道建成通车，安邯快速通道基本竣工，鹤辉高速公路进展顺利。国道341市区改线工程全线开工。全市铁路、高速公路、干线公路网络布局更加完善。加快美丽乡村建设。开展农村环境综合治理，新改建农村公路306公里，新增大中型沼气工程30座，完成新一轮农村电网改造升级任务。实现3.4万农村贫困人口稳定脱贫。实施农村危房改造3030户。新解决38万人饮水安全问题，是比例最高、人数最多的一年。16个乡镇入围全省百强乡镇。

(四)强基础固根本，在提质增收中促进现代农业增效。深入实施现代农业"五大工程"，加快农业结构调整，促进农业增收、农民致富。夯实粮食生产基础。整合农业资金2.9亿元，新建高标准粮田31万亩。加强农田水利基本建设，水库除险加固、大中型灌区续建配套与节水改造等工程进展顺利。提升农业规模效益。新扩建现代农业园区75个。新增农业龙头企业20家，省级农业产业集群达到5个。完成土地流转面积10.5万亩。农民专业合作社达到3124家，家庭农场达到770个。主要农作物农机化水平达到78%。加强生态建设。积极推进林业生态市建设提升工程，营造林20.8万亩，新增城市绿地43万平方米。实施水利项目57个，南水北调总干渠安阳段成功通水，配套工程主体完工。扎实开展地质、气象、测绘、地震等工作，防灾减灾能力不断提高。

(五)促改革扩开放，在重点突破中激发经济发展活力。坚持用改革的办法和开放的途径解决发展难题，发展内生动力和市场活力不断增强。推动重点改革。市级政府机构改革基本完成。全面改进工商登记制度，新增市场主体2.5万个。国企改革"攻坚扫尾"基本完成。有序推进财税、金融改革，扎实开展"营改增"试点工作。县级公立医院综合改革全面启动。农村土地承包经营权确权登记颁证试点工作完成。教育、科技、文化等改革取得阶段性成效。强化开放招商。深入开展招商项目落实专项督察，强力推进赴京与央企对接、"百日攻坚"等重大招商活动，国际家电灯饰产业小镇、枭龙汽车、华能热电联产等项目成功签约。国旅·安阳城、保利影视文化广场、新顺成陶瓷等一批龙头型、基地型、中心性项目落户安阳。外事侨务、对台工作与对外开放相互促进。全年实际利用省外资金481.5亿元，增长16.1%。

(六)强保障惠民生，在和谐建设中提高群众生活水平。坚持把人民利益放在第一位，持续加大民生投入，全市财政民生支出146.9亿元，占财政支出的76.5%。努力扩大就业。深入实施全民技能振兴工程，促进更高质量就业。加强就业困难群体帮扶工作，支持创业带动就业。城镇新增就业7.8万人，农村劳动力新增转移就业4万人，就业困难人员就业8400人。强化社会保障。企业退休人员养老金人均月增200元，实现养老保险各类参保人员在地区之间、制度之间的顺畅转移和衔接。失业、工伤、生育等保险覆盖面继续扩大。城镇居民大病保险试点任务如期完成。全年为城乡低保、农村"五保"等社保对象发放救助资金5.6亿元。开工保障性住房15383套，竣工13467套，5138户群众喜迁新居。全市村级组织换届选举基本完成。依法保护妇女儿童合法权益。社会福利、慈善事业和老龄、红十字、残疾人、关心下一代工作健康发展。大力发展社会事业。优质学前教育资源进一步扩大，4个县(市)获得全省义务教育均衡发展先进县称号，普通高中办学质量有了新提高。职教攻坚二期工程全面启动。高等教育转型发展水平提升。新农合和城镇居民医保财政补助每人每年提高40元。免费为城乡居民提供12类45项基本公共卫生服务。市精神卫生医院投入使用，市人民医院新院区、妇幼保健院儿科病房楼主体完工，医疗资源配置进一步优化。实施"单独两孩"生育政策，促进人口长期均衡发展。公共文化服务体系逐步完善，文化惠民工程深入实施。文物保护工作得到加强。全民健身运动广泛开展，竞技体育取得新成绩。人才、统计、审计、供销、邮政、史志、档案和发展研究取得新进展。维护社会和谐。加强民族宗教工作。强力依法处置非法集资案件。积极化解信访积案，深化安全生产、食品药品安全和社会治安专项治理，确保社会大局稳定。

一年来，我们自觉接受人大法律监督、政协民主监督和社会监督，认真办理人大代表议案、建议和政协提案。深入贯彻全面推进依法治国战略部署，加强民主法治建设，推进依法行政和服务型行政执法，行政效能得到提升。健全权力运行制约和监督体系，反腐倡廉工作取得新成效。军民融合式发展取得新进展，驻安解放军、武警部队和民兵预备役人员为地方发展作出新贡献。深入开展党的群众路线教育实践活动，全面落实中央八项规定精神和省委、省政府20条意见，重点解决"四风"突出问题，全市上下干事创业、务实发展的氛围更加浓厚。

各位代表!

在下行压力大、困难矛盾多的情况下，这些成绩来之不易。这是市委正确领导的结果，是市人大、市政协大力支持的结果，是全市上下团结拼搏、共同努力的结果。在此，我代表市人民政府，向奋斗在全市各条战线上的广大工人、农民、干部、知识分子，向解放军指战员、武警官兵、政法干警以及各界人士，致以崇高的敬意!向所有关心支持我市发展的港澳台同胞、海外侨胞、国际友人，表示诚挚的感谢!

总结成绩的同时，我们清醒认识到，受宏观经济下行、内外矛盾叠加、企业停产限产等诸多因素的影响，经济增速与年度预期目标仍有差距，经济社会发展还存在不少问题和困难。一是下行压力依然较大，钢铁、煤化工等传统支柱行业困难累积，短期内难以走出困境。二是转型任务仍很艰巨，长期形成的粗放型增长方式没有根本扭转，战略性新兴产业和服务业总量较小、比重较低，创新能力不足，农业持续增产、农民持续增收难度加大。三是一些领域体制机制障碍仍然较多，简政放权还不到位，对外开放有待提升。四是城镇化水平不高，民生投入欠账较多，教育、医疗、安全生产、食品药品安全等方面存在薄弱环节。五是潜在风险不容忽视，企业融资难题突出，涉法涉诉信访较多，处置非法集资困难重重。特别是大气污染依然严重，环保压力越来越大。六是政府职能转变不到位，一些部门和公务员能力、素质、作风与经济发展新常态的要求还不适应，一些领域腐败现象时有发生。对此，我们一定高度重视，扎实有效加以解决，绝不辜负全市人民的期望!

二、2015年工作总体要求和主要目标

今年是全面完成"十二五"规划的收官之年。综合判断，世界经济形势依然严峻复杂，整体复苏疲弱态势难有明显改观。我国经济发展进入新常态，增速换挡、结构优化、动力转换的压力增大，但仍处于可以大有作为的重要战略机遇期，总体向好的基本面没有改变。我市与全国、全省一样，在新常态下，投资和要素驱动有所减弱、结构优化升级更加紧迫、资源环境约束加剧、各类潜在风险叠加，但"四化"同步发展为转型升级拓展了空间，改革红利持续释放，区位交通、人力资源、产业集群、载体平台、基础支撑等综合优势更加凸显，全市上下加快转型发展、建设"五个安阳"的态度更坚决，热情更高涨。我们既要增强忧患意识，把困难和挑战估计得更充分一些，更要抢抓发展机遇，坚定必胜信心，顺势而为，乘势而上，奋力夺取转型发展的新胜利!

今年政府工作的总体要求是:全面贯彻党的十八大和十八届三中、四中全会精神，以邓小平理论、"三个代表"重要思想、科学发展观为指导，深入落实市委十届十四次全会部署，主动适应经济发展新常态，坚持转中求进、改革创新，以提高经济发展的质量和效益为中心，更加注重产业转型，更加注重改革开放，更加注重创新驱动，更加注重民生保障，更加注重化解风险，加快打造安阳经济升级版、建设中原经济区区域性中心强市。

经济社会发展主要预期目标是:生产总值增长8%;一般公共预算收入增长9%;全社会固定资产投资增长18%;社会消费品零售总额增长12%;外贸出口增长8%;实际利用外资增长7%;城乡居民收入增速与经济增长同步;人口自然增长率控制在6.4‰以内;居民消费价格涨幅控制在3%左右;节能减排完成省下达任务。

各位代表!

新常态带来新希望，新征程承载新梦想。市委十届十四次全会审议通过了《关于打造安阳经济升级版建设中原经济区区域性中心强市的实施意见》，为我市未来发展绘就了蓝图、明晰了路径。我们要主动适应经济发展新常态，清醒认识增速换挡的必然性、结构调整的复杂性、动力转换的长期性，遵循经济发展规律，主动融入全国、全省区域发展战略新布局，坚定不移贯彻《实施意见》，坚定不移抓好发展第一要务，坚定不移深化改革开放，坚定不移依靠创新驱动，一张蓝图干到底，扭住发展不放松，齐心协力攻难关，谱写安阳改革发展的新篇章!

三、2015年工作安排

今年的任务艰巨而繁重，必须统筹兼顾，抓住重点，把保增长放在更加突出的位置，加快推进"一个载体、四个体系、六大基础"建设。今年着重做好10个方面的工作:

(一)以产业转型为方向，全力建设富强安阳。盯紧需求导向，推动产业向全链条、大集群转型，推动产品向高端化、终端化转型，推动企业向高新技术、现代管理转型，加快建设以先进制造业为基础的新型工业城市。加快改造提升主导产业。综合运用延伸链条、技术改造、兼并重组、淘汰落后等措施，促使主导产业升级。以精密化、智能化、集成化为方向，打造精密制造、液压设备、数控机床等产业集群，做大装备制造产业规模。以控制总量、调整结构、优化布局为思路，加快企业战略重组，发展服务型钢铁、冶金精深加工，推动水泥、陶瓷等产业升级。以拉长链条、弥补短板、循环发展为重点，加快提升工艺装备水平，推动煤化工产业持续发展、循环发展。以龙头带动、壮大规模、集群发展为关键，提升冷链食品、休闲食品、饮料制造、新医药研发等优势产业，增强食品医药产业竞争力。培育壮大基础优势产业。加快纺织服装新城建设，提升纺织服装产业层次。大力承接平板显示、光存储、电器元件、通信产品、软件等产业转移，发展以智能终端为重点的电子信息产业。积极发展以光伏玻璃、光伏电网配套装备为重点的新能源产业。推动建筑业升级，带动能源原材料产业转型脱困。大力发展高成长性产业。积极承接集团化、体系化产业转移，重点支持汽车及零部件、节能环保、家电灯饰等产业成长，努力形成一批以终高端产品为主导的优势产业集群。推进新能源汽车、新型合金材料和超硬材料加速发展。促进产业融合发展。科学谋划和编制产业发展规划，加快信息化工业化深度融合，积极发展云计算、大数据、物联网等，促进新一代信息技术与现代制造业和服务业广泛结合。大力推广智能制造生产模式，加快企业生产设备数字化改造，培育一批数字化车间、智能工厂。打造阿里巴巴安阳产业带。加快建设智慧安阳、无线城市。积极推动企业转型。全面实施"工业转型升级400"。启动铁西老工业区企业整体搬迁，引导企业退城进园。完善企业服务机制、政企沟通机制、银企对接机制、企业权益保护机制和问题受理机制，及时解决企业难题。

(二)以提速增量为重点，推动第三产业发展。坚持生产性服务业与生活性服务业并重，现代服务业与传统服务业并举，进一步提升服务业对经济发展的支撑作用。培育壮大高成长性服务业。提升金融、商务服务、信息服务等产业比重，加大信息技术在现代服务业领域的广泛应用。依托安阳物流港、国旅·安阳城、大通物流园等项目，培育一批大型物流综合园区。启动一批有影响、有带动、有规模的市场建设。提升文化旅游业。围绕建设华夏历史文明传承创新市和国际旅游城，大力实施文化旅游产业"双十工程"。推动红旗渠、太行大峡谷捆绑申报国家5A级景区。完善马氏庄园等景区服务设施，抓好殷墟国家考古遗址公园、曹操高陵文化主题公园、龙泉生态旅游区等开发建设。办好第七届航空运动文化旅游节、"韩国万人游安阳"等活动。支持文化产业加快发展，积极建设特色文化城镇和乡村。大力发展航空经济等新兴产业。加快改造传统服务业。引入现代管理理念和新型商业模式，加快发展电子商务、连锁经营、工厂店等新型业态。坚持控增量、减存量、提质量，促进房地产业健康发展。放宽服务业市场准入。认真落实支持服务业发展的政策，降低医疗卫生、教育培训、健康养老等领域的准入门槛，吸引更多民间资本进入。鼓励县城和中心镇发展特色服务业、涉农服务业和社区服务业。培育城区经济新支撑。增强商务中心区和特色商业区的要素吸附力、产业支撑力、辐射带动力，引导企业、项目、资源要素向"两区"集聚，规划一批区域性综合服务平台，建设辐射周边的地区性服务中心，打造城区发展新的增长点。

(三)以统筹城乡为关键，科学推进新型城镇化。认真落实新型城镇化规划，坚持以推进农业转移人口市民化为重点，实施"一基本两牵动三保障"，促进产业集聚、人口集中、土地集约。优化城镇化空间格局。按照"1+4+1"总体布局，健全空间规划体系，促进中心城区与城市组团产业对接、功能互补，增强县城产业支撑和人口吸纳能力。加快城乡一体化示范区建设，完善基础设施，落实支持示范区发展的政策措施。推动羑河生态景观廊道建设。加强城区建设和管理。以国家卫生城市复审和创建省级文明城市为契机，强化城市环境和建设秩序综合整治，推进精细化、长效化管理。启动永明路南段、文昌大道东段等8条城市主次干道建设。实施安林路、安漳大道等7条道路大修改造工程。打通清风街、富源街等11条"断头路"。完成22条安居配套支路建设。科学规划建设各类管网等地下配套设施。新修建3座人行天桥。积极创建公交优先示范城市。启动城市生活垃圾收运系统升级改造工程。开工建设第八水厂、安钢农贸市场。确保洹北污水处理厂建成投用。加快推进棚户区改造。新启动光明路中段片区、三角湖片区、大营村等22个棚户区和"城中村"改造项目。强力推进苏家村、汪家店等拆迁收尾。开展"城中村"改造政府主导连片开发试点工作。分类推进新农村规划建设。坚持"五规合一"，因地制宜、量力而行，推动新农村综合发展、特色发展。解决25万人安全饮水问题。新改建农村公路300公里。新增沼气用户8000户、大中型沼气工程30座。加快推进农村危房改造。确保2.8万农村贫困人口实现脱贫。积极创建美丽乡村，推进农村人居环境整治。

(四)以转型增效为目标，加快发展现代农业。按照稳粮增收、提质增效、创新驱动的要求，转变农业发展方式，促进农业规模化、集约化、专业化、市场化，加快建设中原经济区现代农业示范市。深入实施高标准粮田"百千万"建设工程。新建高标准粮田30万亩，抓好广润坡10万亩省级示范区建设。粮食产量稳定在218万吨。加快农业产业化集群培育工程。推进"五大基地"建设，促进主要农产品区域化、标准化发展。培育壮大农业龙头企业，推动星河油脂、创新中药材、永达肉鸡等省、市重点集群拉长链条。探索生产要素入股模式，促进集群向农工商一体化发展。力争形成50亿元集群1个、10亿元集群15个。提升现代农业园区发展工程。开展"四级园区"示范创建活动，重点抓好农产品精深加工、内黄都市生态农业、小南海生态长廊、恒大农业、梅平科技等园区建设，增强园区发展效益和辐射带动能力。大力发展都市生态农业，探索精准农业、创意农业、电商农业发展新模式。年内园区面积达到80万亩，设施高效农业面积达到25万亩。实施农业经营体系创新工程。抓好百强示范合作社、百家示范家庭农场创建活动，加快培育新型农业经营主体。推进农业信息化建设，完善农技服务体系，加快农业科技创新和重大技术推广，提升主要农作物全程机械化水平。完善农产品流通服务体系，重点发展一批专业交易市场。实施农业生态保护工程。加快灌区改造，改善农业灌溉条件。大力推广节水、节种、节肥农业新技术，持续推进循环生态农业建设。

(五)以重大项目为带动，努力扩大有效需求。发挥项目拉动作用，促进投资合理增长、结构优化、效益提高。强力抓好项目建设。实施"661"工程，在产业转型升级、重大基础设施建设、先进制造业、高成长性服务业等6个领域，实施600个重大项目，年内完成投资1000亿元以上。重点推进安阳精密制造产业基地、安钢冷轧二期工程、远大可建、中农颖泰、飞凰电动车等工业升级项目。加快建设安阳·中国历史文化产业博览园、周易文化产业园、中华梦幻谷、华强智慧新城等现代服务业项目。扎实推进内黄果蔬商贸城、恒信远大食用菌深加工、中农饲料等现代农业项目。抓好中核汇能、华能热电联产、英利光伏发电等能源项目建设。启动实施安阳市职工文化体育活动中心等社会事业项目。着力扩大投资。围绕农业基础设施、保障性安居工程和农村危房改造、生态建设、社会事业等国家重点支持领域，结合"十三五"规划编制，抓紧谋划实施一批重大项目。争取列入省、市重点项目200个以上。发挥消费基础作用。培育养老健康家政、信息、住房、节能环保、旅游休闲、文化教育体育等一批消费热点。支持建设一批农村超市、城市便民连锁网点。

(六)以强化基础为支撑，不断增创发展优势。把基础支撑能力建设作为增强竞争力的重要组成部分，加快推动局部比较优势向综合竞争优势转变。增强载体平台优势。突出承接转移、集群发展、完善配套、创新机制，推动产业集聚区提质转型创新发展，持续上规模、上层次、上水平，提高吸引力、竞争力、带动力。加快产业集聚区与所在乡镇机构套合。推进专业园区整合重建。强力抓好标准化厂房建设。力争全年产业集聚区固定资产投资增长20%以上。提升基础设施优势。加快实施国道234等5条干线公路新建、改扩建工程。开工建设高铁客运枢纽站、西北绕城高速、新东北外环、安内快速通道。积极推进林桐高速、安阳机场、安阳豫北铁路口岸建设。持续完善信息、能源、生态环境等基础设施。强化要素保障优势。争取各商业银行在信贷规模、业务授权、机构准入、创新试点等方面给予重点支持，吸引金融企业来安设立分支机构。积极培育上市后备企业、挂牌后备企业，支持企业在"新三板"挂牌融资。加大土地收储力度，规范土地交易市场，优化土地资源配置，着力解决批而未征、征而未供、供而未用问题。大力推进"招才引智"，为经济发展提供更有力的人才支撑。

(七)以改革开放为突破，增强发展动力活力。以政府自身革命带动重要领域改革，使改革开放新红利转化为加快发展的新动能。深化行政体制改革。积极跟进中央和省委出台的改革举措。围绕清权、减权、制权、晒权四个关键环节，抓好简政放权，全部取消非行政许可审批事项，大幅度减少前置审批，基本完成市级政府部门权力清单、责任清单公布工作。深入推进国企改革。完善国资监管体制，推进经营性国有资产统一监管。启动国有资本投资公司和运营公司试点、混合所有制经济试点。大力发展非公经济，鼓励非公企业参与国企改革、参与国有资本投资项目。深化财税金融改革。全面规范税收优惠政策。加大财政专项资金清理整合。落实财政资金公开预决算制度。推动融资平台整合，创新政府融资平台管理，优化金融生态环境，防范政府债务风险。设立新型城镇化发展基金、科技创新基金和产业发展基金。加快组建安阳农商行。强化诚信体系建设，建立健全信用信息平台，让"信用"成为市场经济体系的基础桩。按照国家和省部署，推进消费税、资源税等改革。深化农村改革。深入推进农村集体产权制度改革，加快农村土地承包经营权确权登记颁证，开展县级农村产权交易中心试点。规范引导农村土地经营权有序流转，发展多种形式适度规模经营。深化集体林权制度、农电体制改革。同时，完成党政机关公务用车制度改革。积极推进户籍制度、不动产登记、文化体制、价格、社会事业、住房公积金、县级公立医院等改革。创新招商方式。强化企业主体地位。创新开展专题招商、以商招商、中介招商、集群招商，着力招大引强、招新引高。整合一般性的招商活动，推动优惠政策向优化环境转变，全面出击向精准招商转变，增强招商实效。实施本地企业新增投资享受招商引资优惠政策，激励企业再出发、再创业。积极承接产业转移。抓好与保利集团、国旅集团、华强集团、广西黑五类集团等合作项目的跟踪落实。注重承接家居、家电、灯饰、五金、锁具等产业，加快建成一批"小园区大集聚、小企业大产业、小产品大市场"的特色产业园区或小镇，努力形成"众星拱月"好局面。支持企业走出去。鼓励钢铁、水泥等过剩产能行业企业走出去。大力发展对外承包工程与劳务合作。积极引进外向型项目，培育一批外向型产业集群，增加出口带动能力。

(八)以科技创新为驱动，大力建设创新安阳。紧紧抓住科技与经济紧密结合这个核心，大力实施创新驱动发展战略，推动技术创新、产品创新、商业模式创新和市场创新。加强创新合作。突出企业创新主体地位，大力推进科技开放合作，支持企业与国内外同行、大型央企、科研院所、高等院校深度合作。年内实施对外科技合作项目30个以上，引进转化国内外先进技术成果50个以上。提升创新能力。积极实施钢铁、装备制造、节能环保等创新工程，推动传统产业技术工艺创新、信息技术融合。新培育国家级高新技术企业10家以上。新增工程技术中心、研发中心15个。启动实施3个以上重大科技专项和自主创新专项，力争实现产业关键核心技术新突破。健全创新体制机制。完善技术转移和成果转化服务机制，鼓励科技人员创新创业。推动科技成果使用、处置和收益权改革，健全科技人才培养选拔、流动配置、创新激励等机制，让创新活力竞相迸发。加强知识产权保护。实施发明专利倍增计划和知识产权优势培育工程，力争发明专利增长率高于全省平均水平。加快质量强市建设，推进商标战略，推动安阳制造向安阳创造转变。

(九)以环境治理为抓手，加快建设美丽安阳。坚持源头严防、过程严管、后果严惩，建设天蓝、地绿、水净美好家园，让美丽与发展同行。抓好污染防治。深入实施蓝天、碧水、乡村清洁工程，进一步提升环境质量。推动工程减排、结构减排和监管减排，重点强化控煤、控尘、控车、控污、控油"五控"措施，有效遏制雾霾污染。抓好城区河流清洁行动，加强洹河、洪河等水污染治理，推进城区污水处理设施提标改造。开展养殖密集区环境综合整治，推进土壤污染防治和重金属污染修复。按照新《环保法》规定，严厉打击环境违法行为。构建生态文明建设长效机制，努力让环保压力转化为转型动力。改善生态基础。积极推进引岳入安、引红入洹、引漳入羑等项目，畅通城市水系。抓好城乡绿化大提升计划，实施"1+3+N"林业工程，重点抓好森林围城、生态廊道、平原林网、湿地公园和森林公园建设。全年营造林19万亩。集约节约利用能源资源。全面加强节能工作，支持企业实施节能环保技改、新上项目采用先进工艺技术。节约集约用地，推广应用节水节材工艺技术和设备，积极推进园区循环化改造，促进资源利用由高投入、高排放向绿色循环低碳发展转变。

(十)以保障和改善民生为根本，全面建设平安安阳。坚持守住底线、突出重点、完善制度、引导舆论的基本思路，集中财力办好十项民生工程，把钱花在刀刃上。千方百计保持就业稳定。完善就业创业公共服务体系，发挥创业带动就业的倍增效应，重点抓好大学毕业生、农村转移劳动力、城镇困难人员、退役军人等就业。落实失业保险稳岗补贴政策。动态消除"零就业家庭"。促进城乡居民增收。提高城镇中低收入居民收入，完善最低工资标准调整机制。落实机关事业单位人员工资调整方案，实行乡镇工资补贴。多渠道增加农民收入，加大治理欠薪和打击非法中介力度。强化社会保障。城乡居民养老保险基础养老金标准每人每月提高18元，企业退休人员基本养老金提高10%。稳步推进机关事业单位养老保险制度改革。继续提高城乡低保、农村"五保"和优抚对象补助标准。加强社会养老服务体系和县级社会福利中心建设。加大特殊群体帮扶力度，全面实施临时救助制度，兜住保障困难群众基本生活的底线。持续发展老龄、慈善、残疾人、妇女儿童、红十字等事业。加快社会事业发展。加强普惠性幼儿园建设，促进义务教育均衡发展，大力改善农村特别是贫困地区办学条件，推动普通高中多样化、特色化发展。调整城乡学校布局，加强新建城区中小学校建设，力争每个县(市)区新建1所~2所初中、小学，逐步解决"超大班额"问题。大力发展职业教育，建设现代职教体系。支持高等教育转型发展。深化教育教学改革。深入实施文化惠民工程。强化文化遗产的保护和利用。深化文化市场综合执法改革。积极发展档案、史志、发展研究事业。加强基层医疗人才培养和医疗卫生机构标准化建设。新农合和城镇居民医保财政补助标准每人每年提高60元，基本公共卫生服务经费财政补助标准提高到40元。全面实施城乡居民大病保险制度。实施疾病应急救助制度。坚持计划生育基本国策，促进人口长期均衡发展。广泛开展全民健身运动，继续实施老年体育"长青工程"，扎实办好安阳市第十一届运动会。防范化解潜在风险。建立健全化解各类风险的体制机制，强力推进非法集资专项整治，妥善化解困难行业、金融、房地产、社会领域积累的风险，坚决守住不发生区域性、系统性风险的底线。落实依法逐级走访、诉访分离和积案化解制度，切实抓好信访工作。推进社会治理。牢固树立"大平安"理念，着眼"双安"、推进"双治"、强化"双基"，推动社会管理向现代社会治理转变。强化社会治安综合治理，加强安全生产和食品药品安全监管，提升应急处置能力和水平，坚决遏制重特大事故发生，维护社会大局稳定。

广泛开展中国特色社会主义和中国梦宣传教育，大力弘扬焦裕禄精神和红旗渠精神，培育和践行社会主义核心价值观。深入推进公民道德建设，提升全民文明素养，打造文明安阳。加强国防教育和国防后备力量建设，支持驻安解放军、武警部队、民兵和人民防空建设。深入开展"双拥"共建，促进军民融合式发展。加强老区建设。支持工会、共青团、妇联等人民团体发挥桥梁纽带作用。认真做好关心下一代工作。继续做好统计、气象、地震、邮政、外事侨务、民族宗教等工作。积极开展对接京津冀一体化发展等重大专题研究、专案规划、专业推进。科学编制"十三五"规划，坚持长短结合，提高规划的前瞻性和指导性，谋划好重大项目、重大工程、重大生产力布局和重大政策措施，争取一批全局性事项列入国家和省规划，更好地指导发展、引领发展。

四、加强政府自身建设

面对新形势、新目标、新任务和人民群众的新期待，要全面加强政府自身建设，转变政府职能，增强执行力和公信力，建设人民满意政府。

(一)坚持依法行政。把法治理念和法治思维贯穿政府工作全过程，坚持法定职责必须为、法无授权不可为，确保政府工作始终在法治轨道上运行。坚持依法科学民主决策，完善重大决策合法性审查和风险评估机制，推行政府法律顾问制度。加强制度建设，严格用制度管人、管权、管事。自觉接受人大、政协和全社会监督。广泛听取各民主党派、工商联、无党派人士的意见和建议。全面推进政务公开，让人民更好地监督政府，让政府工作更好地体现人民意愿!

(二)大力提升效能。一分部署，九分落实。坚持问题导向、底线思维，务实重干、突出重点、统筹兼顾做好各项工作。强化政府执行力建设，坚决克服"少干事没啥事、多干事怕出事"等消极心态，增强责任担当意识，说了的坚决做到，定了的坚决执行，打通决策落地的"中梗阻"。始终保持群众感情、工作热情和创业激情，以钉钉子的精神狠抓工作落实，少说多做、用心想事、主动干事、努力成事，确保每件事都深入抓、见实效。

(三)持续改进作风。作风建设永远在路上，永远没有休止符。不断巩固和拓展党的群众路线教育实践活动成果，持续深入转变作风，坚决反对"四风"，坚决不搞劳民伤财的形象工程、政绩工程，坚决纠正不作为、乱作为，坚决惩处失职渎职、懒政怠政，努力形成作风建设新常态。加快转变政府职能，强化政府公共服务、市场监管、社会治理、环境保护等职责，切实改进政府服务方式。认真抓好省委巡视组反馈意见的整改落实，问题不解决坚决不松手，整改不到位坚决不收兵。

(四)加强廉政建设。严格执行党风廉政建设责任制和廉洁从政有关规定，加强对项目招投标、工程建设、土地出让、政府采购等重点领域和关键环节的监管。强化行政监察和审计监督，严格规范权力运行。落实"三严三实"要求，以己正立信、以廉政树威、以务实兴业，恪尽职守，践行宗旨，当好人民公仆。厉行勤俭节约，坚决反对浪费。完善惩防体系，坚定不移推进反腐倡廉建设，纠正部门和行业不正之风，努力营造干净干事、风清气正的良好氛围。

各位代表!

全市人民对美好幸福生活的向往，就是我们矢志不渝的奋斗目标。让我们紧密团结在以习近平同志为总书记的党中央周围，在市委的坚强领导下，以踏石留印、抓铁有痕的作风，以敢于担当、苦干实干的精神，以立党为公、公权为民的情怀，抢抓机遇，奋发有为，扎实工作，为全面建成小康社会和中原经济区区域性中心强市而努力奋斗!
